# Volumetric Imaging of Neural Activity by Light Field Microscopy

**DOI:** 10.1007/s12264-022-00923-9

**Published:** 2022-08-08

**Authors:** Lu Bai, Zhenkun Zhang, Lichen Ye, Lin Cong, Yuchen Zhao, Tianlei Zhang, Ziqi Shi, Kai Wang

**Affiliations:** 1 Institute of Neuroscience, State Key Laboratory of Neuroscience, Center for Excellence in Brain Science and Intelligence Technology, Chinese Academy of Sciences, Shanghai, 200031 China; 2 University of Chinese Academy of Sciences, Beijing, 100049 China; 3 Shanghai Center for Brain Science and Brain-Inspired Intelligence Technology, Shanghai, 201210 China

**Keywords:** Light field microscopy, Volumetric imaging, Brain activity, Calcium imaging, Voltage imaging

## Abstract

Recording the highly diverse and dynamic activities in large populations of neurons in behaving animals is crucial for a better understanding of how the brain works. To meet this challenge, extensive efforts have been devoted to developing functional fluorescent indicators and optical imaging techniques to optically monitor neural activity. Indeed, optical imaging potentially has extremely high throughput due to its non-invasive access to large brain regions and capability to sample neurons at high density, but the readout speed, such as the scanning speed in two-photon scanning microscopy, is often limited by various practical considerations. Among different imaging methods, light field microscopy features a highly parallelized 3D fluorescence imaging scheme and therefore promises a novel and faster strategy for functional imaging of neural activity. Here, we briefly review the working principles of various types of light field microscopes and their recent developments and applications in neuroscience studies. We also discuss strategies and considerations of optimizing light field microscopy for different experimental purposes, with illustrative examples in imaging zebrafish and mouse brains.

## Introduction

The brain is densely packed with large populations of neurons that process information at high speed and in a highly parallel manner, in order to cope with the enormously diverse and fast-changing environment. One of the central goals of neuroscience is to understand how such a complex neural substrate and its dynamics represent and process information, as well as give rise to behavioral output. Due to the extremely dense interconnections between neurons, the information-processing flow in the neural network can be better understood if the activities of larger populations of neurons can be monitored simultaneously [1, 2]. Therefore, this has long been the motivation for developing better tools to achieve the recording of neural activities at single-cell resolution and high throughput [2, 3].

Electrical signals of neurons are traditionally monitored by electrophysiology with sharp probes inserted into brain tissue. Due to its invasive nature, the sampling density is usually limited to avoid excessive brain damage. Alternatively, neural activity can also be probed optically if functional molecular indicators can be delivered into neurons and transform various forms of neural dynamics into fluorescence fluctuations. Thanks to the blossoming of chemical dyes and genetically encoded fluorescent proteins in the past few decades, we can now readily label a large population of neurons with molecular specificity to report various functional signals, including Ca^2+^ and K^+^ concentrations, membrane potentials, and many different types of neurotransmitter [4–7]. With all these physiologically interesting signals transformed into optical signals, we can then read them out non-invasively at high resolution and high density with optical imaging techniques in behaving animals. In this scheme, the neural recording throughput is mainly limited by the bandwidth of imaging techniques given that fluorescent indicators are bright and fast. Although many different types of microscopy have been developed for functional neural imaging and each of them is limited in speed by different factors, such as features of light sources, scanning mechanisms, and electronics, the level of parallelization in image collection, which is the number of imaging voxels collected simultaneously, roughly sets a fundamental limit on practical imaging throughput.

According to imaging collection schemes, the various fluorescence imaging techniques can be divided into four major categories: point scanning, line scanning, plane scanning, and scan-free single-shot 3D imaging. Traditional two-photon microscopy and confocal microscopy are point-scanning-based imaging methods and collect information voxel-by-voxel over a 3D volume. The speed of these methods is usually limited by the scanning rate of various scanning mechanisms. Novel techniques of splitting one laser pulse into multiple pulses targeting different locations break the speed limit of two-photon scanning microscopy set by the scanning mechanism [8–11] but the fluorescence lifetime, as well as animals’ tolerance to laser power, still pose a fundamental limit on imaging throughput. To improve imaging speed, line excitation has been applied to sample larger regions simultaneously in two-photon scanning microscopy [12]. These approaches have demonstrated high speed in imaging sparsely-labeled samples over large 3D volumes. A further increase in parallelization of the imaging process can be achieved by light sheet microscopy which combines plane excitation and 2D imaging collection using a fast camera [13]. In this case, scanning is only required in one direction, and the camera’s frame rate usually limits the volume rate depending on the number of planes to collect in an imaging volume.

Eventually, volumetric imaging can be fully parallelized by light field microscopy, which captures 3D images simultaneously in a single camera exposure and without the need for any scanning [14]. The volumetric imaging speed of light field microscopy is only limited by the camera’s maximum frame rate. Benefiting from the recent development of fast complementary metal-oxide-semiconductor (CMOS) cameras, particularly those with large numbers of pixels and highly parallelized electronics, the streaming data rate can ramp up to billions of pixels per second and represents the state-of-the-art of imaging throughput.

Increasing parallelization in imaging collection effectively promotes imaging throughput, but also inevitably raises the risk of inter-voxel crosstalk and makes image collection more vulnerable to tissue scattering and background noise, which are very common in practical imaging applications. Therefore, choosing the right imaging techniques and parameters to balance the strengths and weaknesses of different aspects is crucial for successful imaging experimental design. In this brief review, we introduce the working principles of different types of light field microscopy and the recent advances in improving imaging performance; we discuss the fundamental trade-offs inherent in light field microscopy and strategies for optimizing their performance to meet different challenges; and we summarize successful applications, as well as potential future directions of light field microscopy in neuroscience studies.

## Working Principles of Light Field Microscopy

**Fig. 1:**
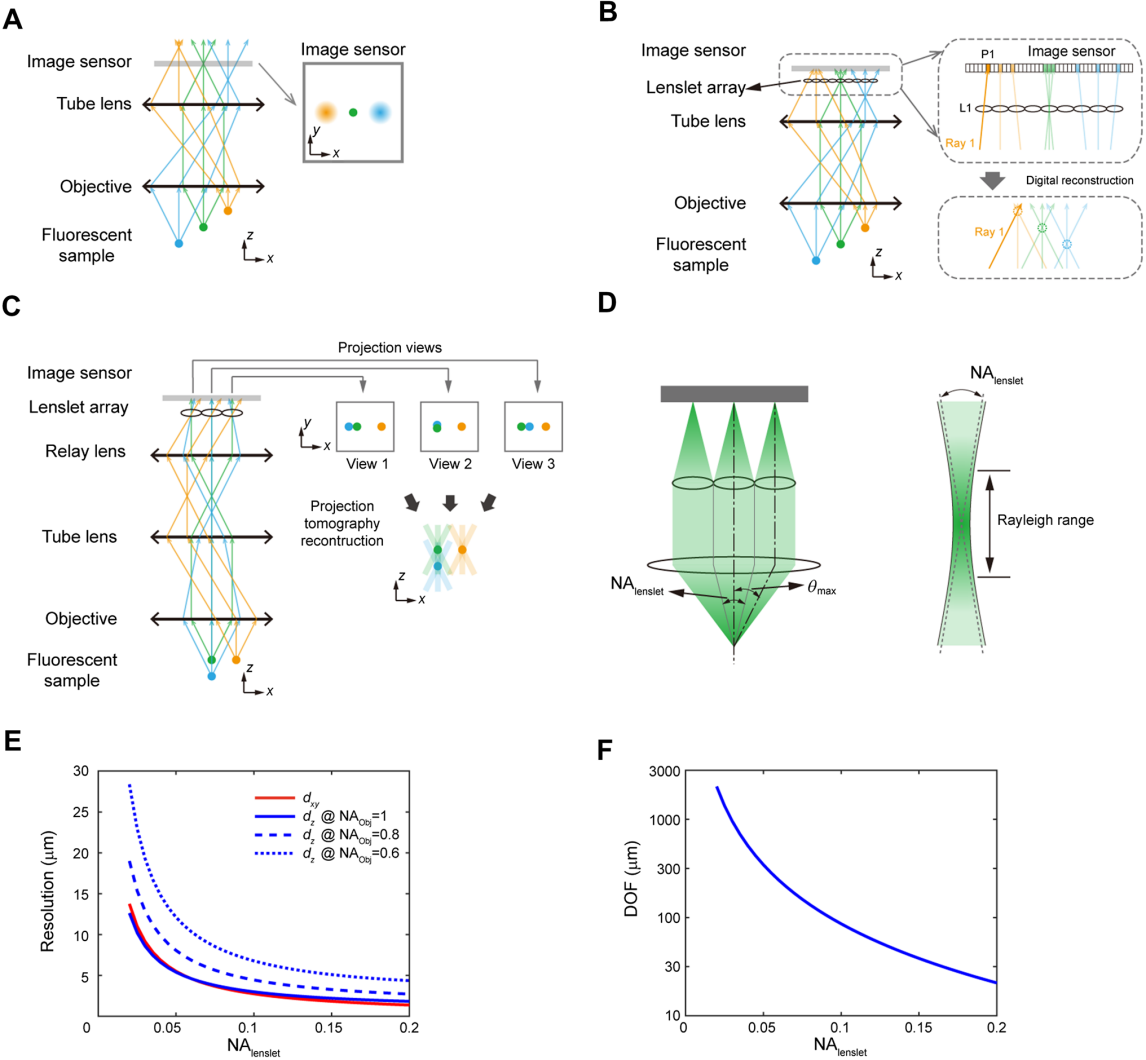
Principles of light field microscopy. **A** Schematics of wide-field microscopy, which forms in-focus (green) and out-of-focus (orange and blue) images simultaneously on the image sensor. **B** Schematics of conventional light field microscopy, which places a lenslet array at the focal plane and moves the image sensor back to the focal plane of the lenslet array. It does not form an image in the conventional sense but captures both positional and directional information of light rays using an image sensor. For example, the positional and directional information of Ray 1 is measured by its intersection with microlens L1 and camera pixel P1. This information is combined computationally to reconstruct a 3D light field, which can then be deconvolved to yield a 3D image, shown as dashed circles. **C** Schematics of Fourier light field microscopy, which places the lenslet array at the pupil plane of the objective by inserting a relay lens after the tube lens. The image sensor captures projection views from each lenslet. These projection views are combined computationally to reconstruct a 3D image. **D** The lenslet array is placed at the pupil plane of the objective in Fourier light field microscopy and each lenslet collects light from a limited NA_lenslet_. The DOF of light field microscopy is estimated as the Rayleigh range of a beam with diverging angle specified by NA_lenslet_. **E** The in-plane resolution of Fourier light field microscopy depends on the NA of each lenslet. The axial resolution depends on both the NA_lenslet_ and the NA_Obj_ of the imaging objective used, where NA_Obj_ is the NA of the imaging objective. **F** The DOF, or the axial coverage, decreases as NA_lenslet_ increases.

When fluorophores are excited in 3D, a traditional optical imaging system actually forms an image of this entire volume simultaneously in 3D imaging space. However, this 3D image is only sampled at one 2D plane by an image sensor in conventional wide-field fluorescence microscopy. Depending on their relative distances from the focal plane, the objects form sharp and blurred images on the image sensor at the same time (Fig. 1A). To achieve simultaneous 3D imaging, multi-focus microscopy splits fluorescent light into multiple beams and focuses them onto different image sensors placed in different focal planes [15]. This approach conserves high resolution and is straightforward to implement, but it requires a complicated optical system and becomes increasingly challenging to scale up to more focal planes. Alternatively, light field microscopy provides an attractive solution to capture 3D volume by a single image sensor with a relatively simple optical system [14].

Different from conventional wide-field microscopy, which essentially only records the locations of light rays intersecting on the 2D image sensor, light field microscopy captures both the positional and directional information of all light rays that comprise the entire light field [16]. The conventional way to achieve this is to place a lenslet array on the imaging plane and move the camera to the focal plane of this lenslet array [14]. In this configuration, each lenslet forms an image of the objective’s back pupil. The lightening of one pixel in this image represents capturing a light ray intersecting both the corresponding lenslet and the camera pixel. Combing information from all pixels, the entire light field consisting of all light rays can be computationally reconstructed and yield a 3D image (Fig. 1B). In a different configuration of light field microscopy, the lenslet array is placed at the objective’s pupil plane [17, 18] and termed Fourier light field microscopy [19]. In this case, light rays collected by each lenslet come from the sample at a certain angle depending on the lenslet’s location on the objective’s back pupil. The images formed by each lenslet on the camera represent projection views of the same sample from different angles. Therefore, the 3D image can be reconstructed from those projection views using algorithms closely related to projection tomography (Fig. 1C).

The ray-optics model described above provides an intuitive picture of how light field microscopy works, but we need an optic-wave model to analyze the achievable resolution across the imaging volume more accurately. Conventional light field microscopy, in which the lenslet array is placed at the imaging plane, has a non-uniform resolution distribution along the axial direction in the imaging volume [20, 21], which depends on a complicated interplay between the angular band limit sampled by each lenslet and the spatial sampling rate of the lenslet array [22, 23]. In contrast, the spatial resolution changes smoothly over the imaging volume in Fourier light field microscopy and can be well understood as the diffraction limit of an imaging system with a reduced numerical aperture (NA) [17]. Hence, we focus on Fourier light field microscopy in the following discussion of resolution and its change across the imaging volume.

## Considerations in Optimizing Light Field Microscopy

### Resolution and Axial Coverage

In Fourier light field microscopy, the in-plane resolution (${{d}}_{{xy}}$) is the same as the resolution of sub-images formed by a lenslet array. Since each lenslet is conjugated to the back pupil of the imaging objective, it only collects light from a limited angular range that corresponds to the NA of the covered sub-region in the objective’s back pupil. This limited angular range sampled by each lenslet is termed ${\text{NA}}_{\text{lenslet}}$, as shown in Fig. 1D. Therefore, the diffraction-limited resolution of the resulting images is given by the Abbe limit [17]:

1 $${{d}}_{{xy}} = \frac{\lambda}{2\cdot{\text{NA}}_{\text{lenslet}}}$$

where *λ* represents the wavelength of the light.

In the axial direction, the resolution depends both on ${{d}}_{{xy}}$ and the maximum light collection angle (*θ*_max_), which is limited by the NA of the imaging objective [17]:

2 $${{d}}_{z} = \frac{{d}_{{xy}}}{{\text{tan}}{\theta}_{\text{max}}}$$

Importantly, the above specified resolution is only maintained near the focal plane of the imaging system with equivalent NA of ${\text{NA}}_{\text{lenslet}}$. In other words, light field microscopy has a limited depth of focus (DOF) determined by ${\text{NA}}_{\text{lenslet}}$. This insight highlights an important trade-off between in-plane resolution ${{d}}_{{xy}}$ and axial coverage DOF in light field microscopy. The DOF can be approximated by the Rayleigh range of a Gaussian beam with divergence specified by ${\text{NA}}_{\text{lenslet}}$ (Fig. 1D) and calculated as [24]:

3 $${\text{DOF}} = \frac{{2\pi}{{d}}_{{xy}}^{2}}{\lambda}$$

According to this equation, light field microscopy has to sacrifice spatial resolution to achieve large axial coverage (Fig. 1E, F). Therefore, it is better suited to image large samples with relaxed resolution requirements rather than pursuing extreme resolution in imaging small specimens. This feature makes light field microscopy an appropriate technique for imaging population neuronal activity at single-cell resolution. For example, we can achieve a resolution of 3.4 × 3.4 × 5 μm^3^ over a volume of more than 200 μm in the axial direction when using an imaging objective with an NA of 1.05 [17].

The trade-off between resolution and axial coverage in light field microscopy can be relaxed by introducing the concept of multi-focus microscopy. In fact, this can be simply achieved by making the focal length of each lenslet in the array different. In this way, each lenslet forms a clear image at different depths, and the combination of information from all lenslet provides high resolution over a concatenated axial range if the sample is not super-densely labeled. This technique has been successfully applied to image neuronal activity over the whole larval zebrafish brain at near single-cell resolution [17].

### Imaging Speed and Photon Budget

In high-speed imaging applications, two types of imaging speed are usually distinguished. One is the instantaneous imaging speed defined as the amount of information that can be recorded simultaneously in a flash of light exposure, the other is the continuous imaging speed representing the temporal resolution. It is very challenging, if not impossible, to optimize both speeds at the same time, but a careful balance between them can yield satisfying performance in many situations.

Light field microscopy is exceptional in achieving a high instantaneous imaging speed because it records 3D volume simultaneously in one camera exposure. The instantaneity is determined by the exposure time, which in turn depends on the camera’s shutter speed or the flash pulse of the fluorescence excitation laser. This feature makes light field microscopy well suited to capture a snapshot of very fast volumetric dynamics without any motion-induced image blur or distortion. In fact, light field microscopy has been successfully applied to imaging neuronal activity over the whole brain in freely-swimming larval zebrafish, the fast-beating heart of larval fish, and circulating blood cells in larval zebrafish and mouse brain [18, 20, 25–27]. Furthermore, the instantaneous imaging throughput can be immediately improved by using cameras with more imaging pixels, which benefits light field imaging *via* enlarging the field of view laterally. For practical Ca^2+^ imaging in behaving animals, such a strategy can also increase throughput because the relatively slow kinetics of Ca^2+^ indicators allows the application of image sensors optimized for reading out frames with extremely large pixel numbers at a reasonable frame rate and low noise.

The continuous imaging speed in light field microscopy solely depends on the maximum allowed frame rate of the camera. If the data transfer rate of the camera is limited, there is a trade-off between the frame rate and the number of pixels per frame, which in turn limits the imaging field of view in light field microscopy. In this scenario, light field microscopy can still be better than other scanning-based volumetric imaging schemes because scanning speed can become a limiting factor in extreme conditions.

High imaging speed usually leads to a reduction in the imaging time span due to a limited photon budget and accelerated photobleaching [17]. However, this trade-off is greatly relaxed in light field imaging due to high light efficiency and low excitation laser peak power. In confocal and two-photon scanning microscopy, increasing imaging speed requires higher peak laser power. Since photobleaching and phototoxicity depend nonlinearly on the excitation laser power, the photon budget is significantly reduced when the imaging speed is high. In contrast, light field microscopy excites all fluorophores in the volume simultaneously and reduces this nonlinear effect to the maximum possible extent [27]. Moreover, light field microscopy has high efficiency because excited fluorescence from all depths is collected non-selectively and contributes to 3D image reconstruction. Therefore, light field microscopy can greatly reconcile the usual conflict between speed and continuous imaging time span. For example, neuronal activity in larval zebrafish during its prey-capture behavior can be imaged by light field microscopy at high quality, indicating that zebrafish larvae can maintain normal physiological functions for a long period under such conditions [17, 24]. The light field microscope can also image the mouse cortex on a time scale of hours with no significant photobleaching [24].

### Background and Optical Sectioning Capability

As introduced earlier, light field microscopy has a limited DOF in which high-resolution imaging can be maintained. The fluorescent signal outside this axial range cannot form a tight focus and essentially becomes an interfering background that degrades the imaging performance. Technically, the background can harm the imaging performance in two different ways. On one hand, the background signal interferes with the reconstruction of the in-focus signal and causes artifacts in the reconstructed 3D volume. On the other hand, the shot noise from the background mixes with the in-focus signal and reduces signal-to-noise ratio (SNR). Therefore, minimizing interfering background is one of the most crucial considerations in optimizing light field microscopy.

The limitation of axial coverage in light field microscopy is set by the requirement of its spatial resolution, as manifested in equation (3). To resolve single cells in the functional imaging of neuronal activity, the spatial resolution has to be kept better than 2 μm–4 μm, depending on the size of neurons in different animal models. This corresponds to a DOF of 50 μm–200 μm. To avoid the background issue, the fluorescence signal has to be confined within this axial range. This is not a big concern when imaging small animals, such as *C. elegans* and larval zebrafish, because the light field microscope can be designed to match its axial coverage to the size of the animal and still provide satisfactory resolution [20]. However, the limited DOF often cannot cover the entire fluorescent labeling in large samples, such as the mouse brain. Therefore, introducing a capability for optical sectioning into light field microscopy is crucial for wider and more flexible applications.

Traditionally, optical sectioning can be introduced either computationally or physically by multiphoton excitation, selective plane illumination, confocal excitation, and detection schemes. Taylor *et al*. devised light field microscopy with computational optical sectioning [25]. It collected multiple images under different excitation conditions sequentially when imaging the mouse brain. They were then computationally combined to yield one image with reduced background. Compared with computational approaches, optical sectioning achieved by physical means not only rejects background, but also removes its inherent shot noise. Yang combined holographic multi-spot two-photon excitation in 3D with light field imaging [28], and Hsu and colleagues achieved localized two-photon excitation in a plane 6.8 μm thick using temporal focusing in the light field [29]. These demonstrations have solved the background issue nicely, but the imaging speeds are limited by the low fluorescence excitation efficiency of two-photon microscopy. Selective one-photon excitation in the desired range can be achieved by illuminating from the side of the sample, similar to light sheet microscopy. This approach in combination with light field microscopy effectively eliminates background and demonstrates high-quality imaging in small animals, especially zebrafish [30, 31]. Due to the restricted optical access from the side of large samples, it is difficult to apply this approach in imaging such samples. The confocal excitation and detection scheme achieves optical sectioning with an epi-illumination configuration and is, therefore, more favorable for large sample imaging, but it is not naturally compatible with light field microscopy. To solve this problem, Zhang *et al*. generalized the traditional confocal detection scheme to make it work in light field microscopy and demonstrated great improvement in functional imaging of neuronal activity in freely-moving larval zebrafish and in mouse brains [24].

## Reconstruction

The raw images captured in light field microscopy contain a mixture of information in a 3D volume and cannot be visualized intuitively. Restoration of 3D image stacks based on raw measurements plays a critical role in achieving optimal performance in light field microscopy and has been under extensive study.

Initially, light field microscopy was adapted from light-field cameras and applied a light ray model for reconstruction [16, 32, 33]. A light ray in the light field $L\left( {x, y,u,v} \right)$ can be defined by the coordinates on the microlens array plane (${u, v}$) and on the camera plane (${x, y}$). Images $I(x, y)$ synthetically refocused at a plane ${f^{\prime}} =\alpha {f}_{0}$, where ${f}_{0}$ is the native focal length and $\alpha$ is a scaling factor that can be changed as needed, and can be calculated from a light-field image parameterized as [32]:

4 $$I(x, y) = \sum_{{u, v}}L[x  + u\left(1- \frac{1}{\alpha}\right), y +  v\left(1-\frac{1}{\alpha}\right), u,v] $$

This synthetic refocusing method can be considered as a summation over different shifted angular views of the sample represented by the light field that the camera records such that the rays forming the views intersect at the desired refocus plane. Synthetically refocused stacks of images are easily generated by changing ${f^{\prime}}$. The reconstruction is straightforward and fast, but it cannot offer high resolution due to the increasing deviations of the light ray model from the actual wave nature of the light at higher resolution. To solve this problem, Broxon *et al*. described a reconstruction method based on the optical-wave model for conventional light field microscopy [22]. By determining the system’s point spread function (PSF), which is the complex diffraction pattern of a point source and takes full consideration of the wave nature of the light, reconstruction with higher resolution can be achieved by applying iterative Richardson-Lucy deconvolution. Because conventional light field microscopy has a spatially varying PSF, this method is computationally demanding. The more accurate modeling of the imaging process yields improved resolution, but this resolution improvement is not uniform across the imaging volume: the limited spatial sampling rate of the lenslet array causes artefact-like low-resolution regions near the native focal plane [22]. A number of new techniques have been developed to reduce these artifacts by coherently combining complementary information obtained from two light field microscopy [31], or introducing structure priors of the imaged samples into the reconstruction algorithm [26, 34]. Nevertheless, the problem of high computational cost remains. Recently, the deep neural network was introduced into 3D image stack reconstruction and demonstrated promising artifact elimination and high reconstruction speed [26, 34]. In this approach, the acquisition of training data is critical for proper training of the neural network. Wang *et al*. generated synthetic light field images using an optical-wave model of light field microscopy and showed that it can generalize properly on actual samples [26]. To get more accurate training data, Wagner *et al.* collected both high-resolution ground truth images and light field images nearly simultaneously on a specialized imaging setup. With both sets of data available, the model can be trained and validated flexibly across the image sessions [34].

Compared with conventional light field microscopy, Fourier light field microscopy offers two advantages in image reconstruction [17]. First, the imaging process can be conveniently described by a spatially invariant PSF, which greatly reduces the computational cost. The forward imaging process can be described as [17]:

5 $${{I}} = \sum_{{z}}{{\text{Object}}}_{{z}} {\otimes} {\text{PSF}}_{{z}}$$

At each axial location *Z*, the contribution to the imaging is simply a convolution between ${\text{Object}}_{{z}}$ and the spatially invariant ${\text{PSF}}_{{z}}$. The reconstruction can also be achieved by Richardson-Lucy deconvolution, but at greatly reduced computational cost due to the spatially invariant PSF. Second, the optical system physically guarantees a uniform resolution across the imaging volume. In this case, optical-wave-model-based reconstruction demonstrated artifact-free high-resolution imaging with high fidelity, and at moderate computational cost [17]. Deep-learning-based reconstruction has not been applied to Fourier light field microscopy, but it potentially can achieve a higher reconstruction speed in the same way demonstrated in conventional light field microscopy.

Specialized reconstruction methods can be devised for specific imaging applications. For example, Nöbauer and colleagues developed an algorithm to extract Ca^2+^ traces of active neurons when imaging population neuronal activity in the mouse brain [35]. By making use of the fact that the images of neurons only have intensity fluctuations and do not change their positions, the algorithm can be tailored to speed up greatly by mitigating the need to reconstruct every single frame separately. Yoon *et al*. also made use of the spatial-temporal sparsity in neuronal activity to achieve better performance in the functional imaging of Ca^2+^ transients [36].

## Discussion

Light field microscopy is distinguished from conventional fluorescence microscopy by providing an instantaneous 3D imaging capability. Recent technological developments have continuously improved its performance in resolution, background rejection, reconstruction quality, and speed, but its application in biological studies is still in its infancy. Here, we summarize some exemplary applications that benefit from the unique features of light field microscopy and discuss their future directions.

**Fig. 2:**
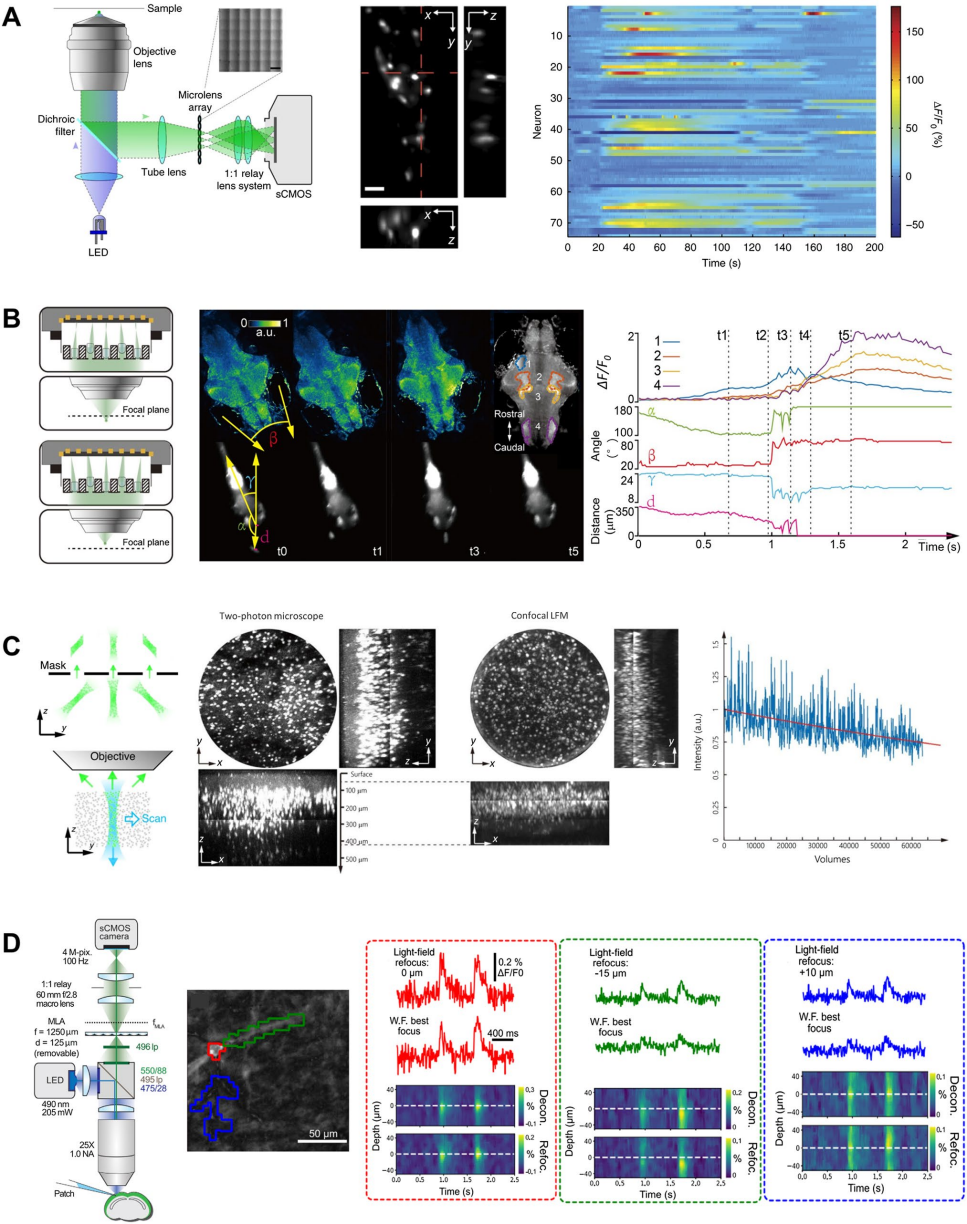
Applications of light field microscopy in neuroscience. **A** Imaging neuronal activity in small animals by conventional light field microscopy. Left, schematics of traditional light field microscopy (scale bar, 150 μm). Middle and right, example images of *C. elegans* and its neuronal activity traces captured by light field microscopy (scale bar, 10 μm) (adapted with permission from Springer Nature [20]). **B** Light field imaging of freely-swimming larval zebrafish. Left, schematics of extended field-of-view light field microscopy. Middle, whole-brain functional imaging during prey capture behavior of larval zebrafish. Right, quantification of larval zebrafish behavior and neuronal dynamics during a successful prey capture of a paramecium (adapted with permission from Cong *et al.*, 2017 [17]). **C** Volumetric imaging of the mouse brain by confocal light field microscopy. Left, concept of generalized confocal detection. Middle, confocal light field microscopy captures most of the neurons within ~400 μm deep into the mouse cortex, as confirmed by two-photon microscopy. Right, confocal light field microscopy captures >50,000 volumes without causing significant photobleaching (adapted with permission from Springer Nature [24]). **D** Voltage imaging of dendritic branches in acute mouse brain slices by light field microscopy. Left, schematic of light field microscopy optimized in resolution and imaging speed for voltage imaging; Middle, example regions of interests (ROIs) in light field imaging; Right, extracted voltage signals in these ROIs. sCMOS, scientific complementary metal-oxide-semiconductor; LFM, light field microscopy; MLA, micro-lens array; W.F., wide field; Decon., deconvolved; Refoc., refocused (adapted with permission from Quicke *et al*., 2020 [45]).

Early demonstrations of light field microscopy in neuroscience were performed on small animals, including *Caenorhabditis elegans*, larval zebrafish, and *Drosophila* [20, 37]. Due to its small size, a light field microscope can be designed to image the entire brain of *Drosophila* and zebrafish larvae (Fig. 2A) or the whole body of *C. elegans*. Compared to competing imaging techniques, such as light sheet microscopy, light field microscopy offers higher speed, but it has a relatively low resolution that cannot reliably resolve single neurons. To resolve this problem, a line of research has been devoted to improving resolution and imaging performance [17, 22, 37]. On the other hand, light field microscopy has also found new applications in imaging neuronal activity over the whole brain in freely-moving animals. Many neuroscience studies have favored research on freely-moving animals [38] because rich and natural behaviors are better preserved. However, functional imaging is conventionally achieved on movement-restrained animals due to the great technical difficulties. Fortunately, the instantaneous 3D imaging capability provided by light field microscopy readily resolves many challenges. Cong and colleagues integrated light field microscopy with a 3D fast-tracking system and achieved imaging of whole-brain neuronal activity at near single-neuron resolution in freely-swimming larval zebrafish [17, 24]. The motion-induced image blur can be kept at a sub-micron level even when zebrafish larvae move as fast as 6 mm/s by applying very brief excitation laser exposure. With the new experimental paradigm (Fig. 2B), previously challenging studies, such as the prey capture behavior of larval zebrafish, can be performed at unprecedented resolution. In the future, these technical advances can potentially benefit many more interesting studies, such as social behaviors in freely-moving animals.

Extending light field imaging to larger animals, such as mice, could find wider applications in neuroscience studies. At the same time, the increased sample size raises many technical difficulties, including tissue scattering and background interference. Several strategies have been developed to meet these challenges. For example, Nöbauer and colleagues developed a reconstruction algorithm specifically tailored for Ca^2+^ imaging in scattering mouse brains to increase the SNR and reduce computational cost [35]. Taylor *et al*. introduced computational optical sectioning into light field imaging to reduce interfering background [25]. In addition, Zhang *et al*. proposed a generalized confocal detection scheme (Fig. 2C) to both reduce the background and improve the SNR [24]. Besides innovations in optics and data processing, the development of new fluorescent indicators and labeling techniques can also effectively improve the imaging performance of light field microscopy. For example, reduced tissue scattering and enhanced tissue penetration depth can be expected if Ca^2+^ indicators with longer emission wavelengths are applied in light field imaging [39]. Localizing Ca^2+^ indicators to regions near neuronal somas can improve the SNR by reducing neuropil contamination in the same way as demonstrated in wide-field imaging [40, 41]. These collective advances make light field microscopy an attractive solution for large-scale functional imaging in large animals.

Besides imaging neural activities reflected by Ca^2+^ transients, the high imaging speed of light field microscopy potentially can have a major impact on capturing even faster dynamics of neuron membrane potentials. Unlike Ca^2+^ imaging, which is limited in speed to resolve fast spike trains and provides little information on subthreshold neuronal computations, voltage imaging can report membrane potential changes faithfully and at unprecedented temporal resolution [42, 43]. However, the fast dynamics and relatively low sensitivity of currently-available voltage indicators make voltage imaging much more challenging than the widely applied Ca^2+^ imaging [44]. In this case, light field microscopy can be more advantageous in imaging voltages in a larger 3D volume and across a longer period of time than conventional scanning two-photon microscopy because it achieves high imaging speed at lower peak power of the excitation laser. In an early attempt, Quicke and colleagues demonstrated subcellular resolution genetically encoded voltage indicator (GEVI) light-field imaging in acute mouse brain slices (Fig. 2D) resolving dendritic voltage signals in 3D [45]. This strategy can be extended to achieve population voltage imaging by covering larger volumes at a relaxed resolution to resolve single neurons. In fact, several recent studies have demonstrated *in vivo* wide-field imaging of newly-developed voltage indicators that are localized in the soma and offer higher sensitivity and better photostability in mouse brains [7, 46]. We anticipate improved voltage imaging by combining these indictors with light field microscopy because the interfering background in wide-field imaging can be converted into useful 3D imaging and consequently achieves higher imaging throughput at better SNR.

Although the very original idea of light field microscopy can be traced back to the light field camera invented about a century ago, its recent renaissance relies critically on collective advances in the high-speed digital camera with large pixel numbers, computational power provided by modern computers, or even graphic processing units and advanced mathematical tools, as well as wider applications found in biological imaging. On one hand, the rapid technological developments in optics and electronics will undoubtedly further enhance the capability of light field microscopy. On the other hand, more diverse functions can be motivated by its continuous expanding palette of biological imaging applications, particularly in neuroscience studies, and make light field imaging a critical and flexible solution complementary to conventional confocal microscopy, two-photon microscopy, and light sheet microscopy.
